# Water and Molecular Exchange in Biological Cells Studied Using ^1^H Pulsed Field Gradient NMR

**DOI:** 10.3390/membranes13060567

**Published:** 2023-05-30

**Authors:** Irina A. Avilova, Vitaly I. Volkov

**Affiliations:** 1 Federal Research Center of Problems of Chemical Physics and Medicinal Chemistry RAS, 142432 Chernogolovka, Russia; vitwolf@mail.ru; 2 Scientific Center in Chernogolovka, The Institute of Solid State Physics RAS, 142432 Chernogolovka, Russia

**Keywords:** biological cell, biological membrane, pulsed field gradient NMR, diffusion, permeability, lipid bilayers, lateral diffusion

## Abstract

This review presents the results of studies of molecular exchange processes in various biological systems (erythrocytes, yeast, liposomes, etc.) performed using pulsed field gradient NMR (PFG NMR). The main theory of processing necessary for the analysis of experimental data is briefly presented: the extraction of self-diffusion coefficients, calculation of cell sizes, and permeability of cell membranes. Attention is paid to the results of assessing the permeability of biological membranes for water molecules and biologically active compounds. The results for other systems are also presented: yeast, chlorella, and plant cells. The results of studies of the lateral diffusion of lipid and cholesterol molecules in model bilayers are also presented.

## 1. The Concept of a Biological Membrane

The elementary structural unit of all living systems is the cell—an integral system containing many interconnected elements (organelles) [1]. The contents of the cell are separated from the external environment by a cell membrane or biological membrane (biomembrane), which ensures its integrity and regulates metabolism. The following are according to the fluid mosaic model of biomembrane organization proposed by S. D. Singer and G. L. Nicholson in 1972 and still used [2]:

- The structural basis of biomembranes is a lipid bilayer, in which the hydrocarbon chains of phospholipid molecules are in a liquid-crystalline state.
- The lipid bilayer, which has the viscosity of vegetable oil, is immersed or embedded with protein molecules that can move across the membrane.

According to modern concepts, the cell membrane is a molecular structure consisting of lipids, proteins, and carbohydrates. It separates the contents of any cell from the external environment, thereby ensuring its integrity, and it regulates the metabolism between the cell and the environment [1]. The distribution of the components of the cell membrane is asymmetric, i.e., different in the outer and inner layers of the membrane. Such asymmetry determines the existence of lateral diffusion—the self-diffusion of lipid molecules in the plane of the bilayer, which is associated with many characteristics of biomembranes (fluidity, orderliness, and permeability) [3]. As a rule, such studies are performed on model biological membranes, which are mono- or bi-layer lipid vesicles.

Since a cell is a thermodynamically open system that continuously exchanges matter and energy with the environment, the transport of substances through biological membranes is a necessary condition for life. The most important property of a biomembrane is selective permeability, i.e., the ability to pass some substances and not pass others [4]. The transfer of substances through biomembranes is associated with the processes of cell metabolism, bioenergetic processes, the generation of a nerve impulse, etc. The processes of membrane transfer are usually divided into two main types: passive and active.

Via active transport, a substance is transferred through a biological membrane against a concentration gradient (from a region of low concentration to a region of high concentration), i.e., with the expenditure of free energy.

The transfer of a substance along a concentration gradient from a region of high to a region of low concentration without energy expenditure is carried out via passive transport (osmosis and diffusion). The methods of diffusion transfer are quite diverse: the diffusion of fat-soluble substances through the lipid part of the membrane; the transport of hydrophilic substances through the pores formed by membrane lipids and proteins; and facilitated diffusion involving special carrier molecules.

Non-polar substances, such as organic and fatty acids, and esters, easily dissolve in the lipid phase of the membrane due to their increased affinity for the lipid bilayer. Polar substances (inorganic salts and sugars) do not pass well through the lipid bilayer.

At the same time, for water molecules, the permeability of biomembranes is about 10^−6^ m/s, which is quite large for a polar substance that is insoluble in lipids [5]. The explanation for this phenomenon was found after the discovery of integral membrane proteins, called “aquaporins” [6]. These molecules form pores in cell membranes through which water molecules selectively pass. At the same time, they do not allow ions and other soluble substances to pass through. Thus, there are two ways for water molecules to enter a cell: through the lipid bilayer and through aquaporins. The interest in the permeability of biomembranes for water molecules is due to a number of functions that water performs: it is a solvent for many substances; it acts as a medium for chemical reactions and participates in them (hydrolysis); and it maintains the shape of the cell and is involved in its thermoregulation.

In the study of the permeability of biomembranes for various compounds, a number of physicochemical methods are used: osmotic, based on measuring the change in cell volume when placed in hyper- or hypo-tonic solutions; indicator, based on a change in the color of the cellular contents with the introduction of certain substances; chemical, based on the analysis of the composition of intracellular contents; and the method of labeled atoms, based on the use of radioactive and stable isotopes, among others [7]. A wide range of opportunities for the non-invasive study of local molecular mobility and molecular diffusion in living biological cells is provided by the pulsed field gradient NMR method (PFG NMR) [8].

## 2. Pulsed Field Gradient NMR in the Study of Biological Systems

Since the PFG NMR method is not as widely used as other NMR methods, this section is devoted to an introduction to the basics of the method. The uniqueness of the PFG NMR method lies in the possibility of the direct measurement of the self-diffusion coefficient and the relative proportions (populations) of diffusant molecules in various phases of heterogeneous systems. The non-invasiveness of the method allows it to be used to study diffusion processes and evaluate permeability in biological membranes.

**Figure 1:**
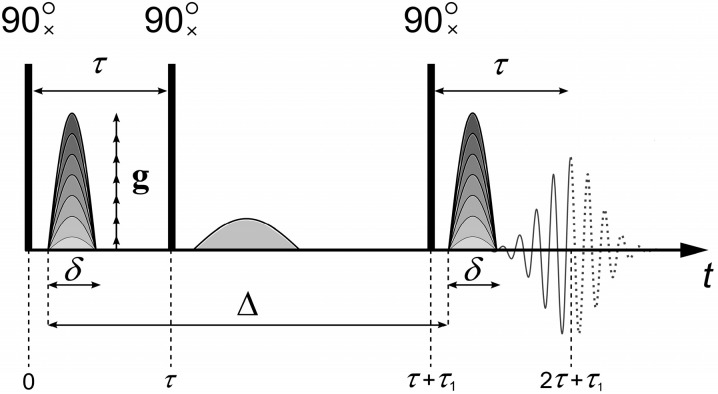
Three-pulse “stimulated echo” sequence with a pulsed magnetic field gradient: *δ* is the duration of the pulsed magnetic field gradient, *g* is the amplitude of the pulsed magnetic field gradient, *τ* and *τ*_1_ are the intervals between radio frequency (RF) pulses, Δ is the interval between the pulses of the magnetic field gradient.

To measure the diffusion coefficients, the “stimulated echo” sequence shown in Figure 1 is used [9,10,11].

The stimulated echo sequence consists of three radio frequency (RF) 90° pulses and two magnetic field gradient *g* pulses (Figure 1). The main parameters that are set in the experiment are the duration of the pulsed magnetic field gradient *δ*, the amplitude of the pulsed magnetic field gradient *g*, and the interval between the pulses of the magnetic field gradient Δ. The magnetic field gradient pulses are applied between the first and second, as well as after the third, RF pulses. The first pulse of the magnetic field gradient encodes the position of the spin ensemble in space. The second pulse of the magnetic field gradient, applied after time Δ, decodes the position of the spin ensemble. Under the experimental conditions, the first gradient *g* causes a dephasing of the magnetization vector $\vec{M}$, which the second gradient *g* revokes depending on the spatial motion of the molecules.

**Figure 2:**
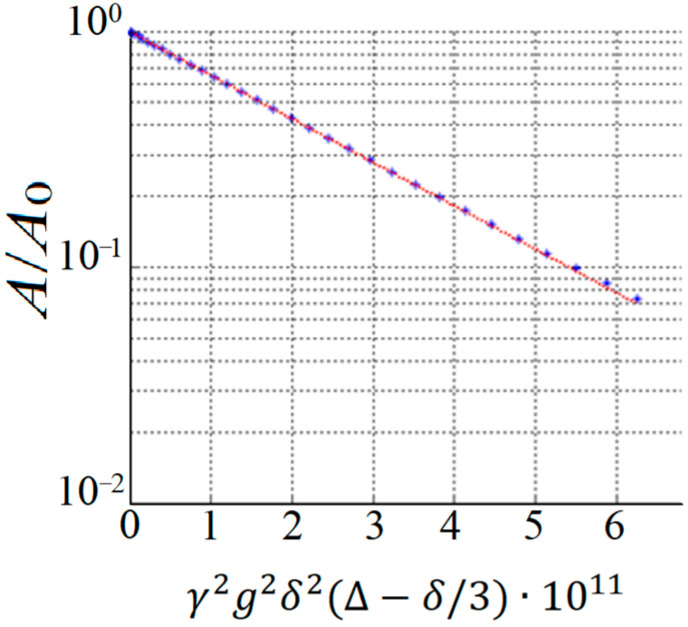
An example of DD obtained as a result of a PFG NMR experiment.

By performing a PFG NMR experiment, the diffusion decay (DD) is obtained, which represents the dependence of the amplitude of the spin-echo signal *A* on the parameters of the magnetic pulse gradient. In this case, one of the parameters, *g*, *δ*, or Δ, is varied. An example of exponential diffusion decay is shown in Figure 2.

In the case of the isotropic Brownian motion of diffusant molecules, the resulting diffusion decay (Figure 2) is described using Equation [9]:

(1) $$A\left(2\tau ,\;\tau_{1},g\right)=A\left(2\tau ,\;\tau_{1},0\right)exp\left(-\gamma^{2}g^{2}\delta^{2}t_{d}D_{s}\right),\;A\left(2\tau ,\;\tau_{1},0\right)=\frac{A\left(0\right)}{2}exp\left(-\frac{2\tau }{T_{2}}-\frac{\tau_{1}}{T_{1}}\right)$$

where *A*(0) is the NMR signal amplitude measured immediately after the 90° pulse; *T*_2_ is the spin–spin relaxation time; *T*_1_ is the spin–lattice relaxation time; γ is the gyromagnetic ratio; *t_d_* = ∆ − 1/3*δ* is the diffusion time; *D_s_* is the self-diffusion coefficient (SDC); and *g*, *δ*, *τ*, and *τ*_1_ are shown in Figure 1. When measuring the evolution of the echo signal, *τ* and *τ*_1_ are fixed, and only the dependence of *A* on g is analyzed.

For multiphase systems, Equation (1) becomes more complicated. In NMR, the phase of a system is the region in which the molecules are characterized by the same SDC and relaxation times. In this case, the equation describing the diffusion decay in the system under study has the following form [12]:

(2) $$A\left(g\right)=\frac{A\left(2\tau ,\tau_{1},g\right)}{A\left(2\tau ,\tau_{1},0\right)}=\sum_{i=1}^{m}p_{i}^{'}exp(-\gamma^{2}g^{2}\delta^{2}t_{d}D_{si}),\;p_{i}^{'}=p_{i}exp\left(-\frac{2\tau }{T_{2i}}-\frac{\tau_{1}}{T_{1i}}\right)/\overset{m}{\underset{i=1}{\sum }}p_{i}exp\left(-\frac{2\tau }{T_{2i}}-\frac{\tau_{i}}{T_{1i}}\right),\;\sum_{i=1}^{m}p_{i}=1,$$

where *D_si_* is the self-diffusion coefficient in the *i*-th region (phase), and pi is the relative fraction of the molecules in the *i*-th region (the population of the *i*-th phase).

By analyzing the dependence *A*(*g*) under the conditions of slow exchange, the partial self-diffusion coefficients *D_si_* and the relative shares pi of diffusant molecules in various nano- and micro-volumes of membrane systems are determined. Modern NMR spectrometers make it possible to record spin-echo spectra with a Fourier transform, which makes it possible to measure the partial self-diffusion coefficients of various molecules or fragments of molecules in the range from 10^−14^ to 10^−8^ m^2^/s. The use of the Fourier transform of the spin-echo spectra makes it possible to obtain high-resolution NMR spectra of the systems under study, which greatly simplifies the interpretation of diffusion data. For example, it allows one to perform a correlation between lines in the spectrum and molecules in the system under study.

**Figure 3:**
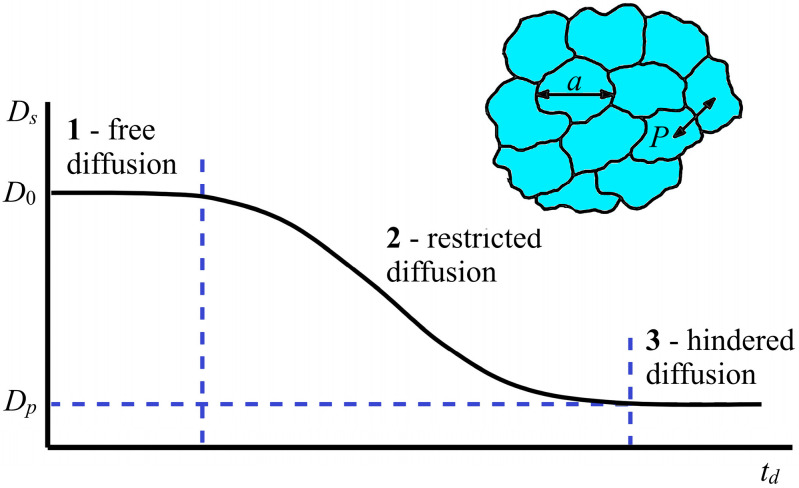
Idealized dependence of the self-diffusion coefficient *D_s_* of diffusant molecules on the diffusion time *t_d_* in porous systems with permeable walls. *D_0_* is the coefficient of free diffusion, *D_p_* is the coefficient of limited diffusion, a is the size of the restricted area (pore or cell diameter), *a* = (6*Dt_d_*)*1*/2; *P*—pore (cell) permeability [13].

For porous systems, which can be considered models of biological cells, it is possible to estimate their size and permeability. In this case, the dependence of the SDC *D_s_* on the diffusion time *t_d_* is analyzed under the conditions of the limited diffusion of water in the microvolume of membrane systems [13,14,15,16,17,18,19]. There are three modes of the dependence of the SDC *D_s_* on the diffusion time *t_d_*: short-term, intermediate, and long-term dependence. The ideal form of such a dependence is shown in Figure 3.

As can be seen in Figure 3, at short diffusion times *t_d_* (region 1), the diffusant molecules do not reach the pore walls, and a self-diffusion coefficient *D_s_* = *D*_0_ does not depend on td; *D*_0_ is the self-diffusion coefficient of the bulk liquid.

In the area of restricted diffusion (Figure 3, region 2), a large number of diffusant molecules collide with the pore wall, and *D_s_* depends on *t_d_*, becoming a decreasing function of time.

At long diffusion times *t_d_*, for the diffusant molecules, as a result of the penetration effect, to diffuse into neighboring pores, their movement is averaged over the entire pore space; the measured diffusion coefficient *D_s_* = *D_p_* also does not depend on td (Figure 3, region 3), and its value is less than the volume *D*_0_. If self-diffusion is observed in a completely isolated pore, then the root-mean-square displacement of molecules is limited by the pore size. Therefore, as the diffusion time increases, the SDC *D_s_* decreases. In the limit *t_d_*→∞, the SDC *D_s_* tends to be zero. A sign of a long-term regime for isolated pores is the fulfillment of the dependence *D_s_* ∝ $t_{d}^{-1}$—completely limited diffusion. From the data for *D_s_* in this mode, the linear dimensions of isolated pores *a* are estimated in accordance with the Einstein relation:

(3) $$D_{s}\left(t_{d}\right)=\frac{\langle a^{2}\rangle }{6t_{d}},$$

where *a* is the pore size.

Since in permeable systems (biological cells) there is no mode of completely limited diffusion, i.e., dependence *D_s_*(*t_d_*) ∝ $t_{d}^{-n}$, where *n* < 1, it is impossible to directly use Equation (3) to determine the pore size *a*. This situation was considered in detail in [14,16,19], where the scaling approach proposed by Skirda V.D. et al. was used to analyze the *D_s_*(*t_d_*) dependence. In this case, the effective SDC $D_{s}^{eff}$ is calculated, the dependence of which on the diffusion time *t_d_* is similar to that observed for systems with closed pores, i.e., ∝$t_{d}^{-1}$, which allows for the use of $D_{s}^{eff}$ to estimate the size of permeable pores:

(4) $$D_{s}^{eff}\left(t_{d}\right)=\frac{D_{s}\left(t_{d}\right)-D_{p}}{D_{0}-D_{s}\left(t_{d}\right)}\cdot D_{0},$$

where *D*_0_ is the SDC of the bulk liquid; i.e., in the absence of restrictions (*t_d_*→0), *D_s_*(*t_d_*) is the experimentally obtained dependence, and *D_p_* is the SDC of diffusant molecules in the long-term diffusion regime, i.e., in the mode of averaging over the entire porous space (*t_d_*→∞).

If the values of *D*_0_, *D_p_*, and the pore size *a* are known, then the permeability of the pore wall *P* can be calculated from the ratio obtained on the basis of the analysis of the resistance to the transfer of diffusant molecules through a number of parallel permeable barriers (membranes) separated by distance *a* (cell size) [20]:

(5) $$\frac{1}{D_{p}}=\frac{1}{D_{0}}+\frac{1}{P\cdot a},$$

where *P* is the permeability of the pore wall, and *a* is the pore size.

Equation (5) does not take into account the details of the mechanism of intercellular diffusion. As mentioned earlier, water molecules enter the cell in two ways: through the lipid bilayer and aquaporins. Therefore, Equation (5) can only be used to assess the total permeability of biological membranes.

Since the cell wall is permeable, there is a molecular exchange between the water inside the cell and the water in the outer volume. To estimate the residence time of a water molecule inside a cell, a two-component model of molecular exchange is applied. From this model, a relative fraction *p* of the diffusant molecules in a cell depends on the diffusion time *t_d_* as follows [12]:

(6) $$p\left(t_{d}\right)=p\left(0\right)exp\left(-\frac{t_{d}}{\tau }\right),$$

where *τ* is the “lifetime” of the molecule inside the cell.

Thus, the analysis and interpretation of data obtained via PFG NMR make it possible to determine such important cell parameters as the permeability of biomembranes for various substances, in particular for water molecules, to estimate the size of cells and the residence time of diffusant molecules inside the cell. The following sections present the results obtained in this field for various biological systems.

## 3. Studies of the Permeability of the Erythrocyte Membrane

An erythrocyte (from the Greek *erythros*—red) is a biconcave disk-shaped blood cell, the main function of which is the transport of gases in blood [21]. Erythrocytes (red blood cells—RBCs) are of particular interest for studying the structure of the biological membrane and the transport of substances through erythrocytes. Firstly, RBCs are the main component of blood (the volume fraction in the blood is 40–45%). They play a key role in the formation of the rheological parameters of blood; i.e., they determine its fluidity. Secondly, the erythrocyte membrane is inherent in the general principles of the molecular organization of the biomembrane. This makes it possible to extrapolate patterns of structural and functional changes in the erythrocyte membrane to other membrane systems with certain corrections.

The shape of the erythrocyte cell causes a large ratio of cell surface area to its volume, which ensures a rapid exchange between the cell and the environment. The main content is hemoglobin, a protein that reversibly binds to oxygen [22]. The functions performed by erythrocytes in the body are diverse: gas transport (oxygen transfer); buffer (pH regulation); nutritional (the transfer of amino acids and glucose); protective (the adsorption of toxic substances), etc.

**Figure 4:**
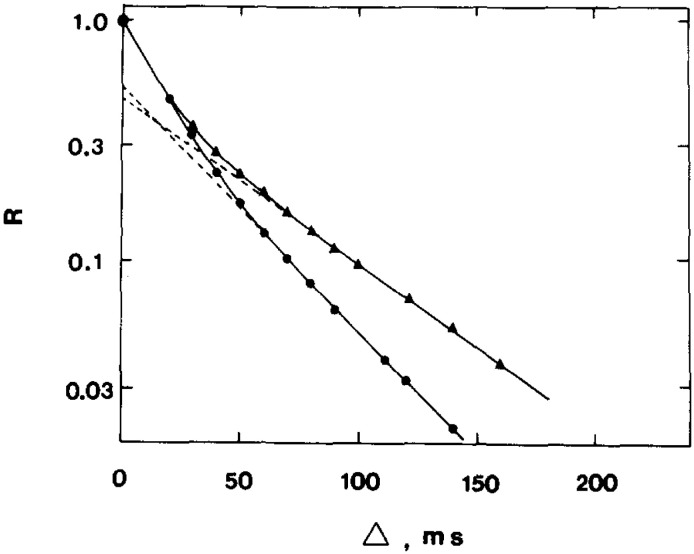
The echo amplitude for water protons in human blood as a function of ∆ at 24 °C. The measurements were carried out on pure blood (●⸺●) and blood with 20 mM *p*-chloromercuribenzoate added to the plasma (▲⸺▲). The experimental constant *K* was 2.03 × 10^6^ cm^−2^. The dashed lines represent extrapolations used to obtain the diffusion coefficient *D*_1_ [23].

The method of PFG NMR has found application in the study of the permeability of the erythrocyte membrane [23,24,25,26,27,28,29,30,31,32,33,34,35,36,37,38,39,40]. In what follows, some of the most interesting results, in our opinion, are presented. The authors of the work [23] conducted a study of the permeability of the human erythrocyte membrane for water molecules and evaluated the effect of the reagent para-chloromercuribenzoate (pCMBS), which inhibits the osmotic permeability of water, via the average lifetime of water in erythrocytes. The work demonstrated that the addition of para-chloromercuribenzoate to blood plasma leads to a change in the form of the diffusion decays of the protons of water molecules in a suspension of erythrocytes (Figure 4).

From the experimental data, it was found that the addition of p-chloromercuribenzoate to blood plasma leads to a significant decrease in the rate of water exchange and, accordingly, to an increase in the “lifetime” of a water molecule inside the cell. In pure blood plasma, the value of *τ* was 17.0 ms, and after the addition of p-chloromercuribenzoate, *τ* = 48.0 ms. The results obtained even before the discovery of aquaporin demonstrated that there are at least two ways for the diffusion of water molecules into the erythrocyte.

A number of important functions in the body are performed by the hemoglobin contained in erythrocytes [24]. Hemoglobin is a two-way respiratory carrier that transports oxygen from the lungs to tissues and facilitates the reverse transport of carbon dioxide [25]. In the work [26], the diffusion coefficients of oxyhemoglobin A (HbA-O2) and oxyhemoglobin S (HbS-O2) in intact erythrocytes were measured using PFG NMR. The authors demonstrated the possibility of measuring the diffusion coefficients of hemoglobin not only in a solution but also directly in erythrocytes. A comparative analysis of the diffusion coefficients of HbA-0 and HbS-0 in erythrocytes under similar conditions showed that the *D* values for HbS-0 are 10% lower than those for HbA-4. It was also shown that the apparent diffusion coefficient for intracellular water molecules is an order of magnitude smaller than that for bulk water. In [27], the diffusion of water in human and camel erythrocytes was compared. As a result, it was found that the apparent diffusion coefficient of the water molecules in camel erythrocytes is 15% lower than in humans. Experimental data led to the conclusion that a lower apparent diffusion coefficient is associated with a more pronounced increase in the osmotically inactive water fraction. It is assumed that the increased hydrophilicity of hemoglobin allows not only for increased water binding but also for hemoglobin to be packed more tightly in vivo, which, in turn, is associated with a slower apparent diffusion coefficient and increased osmotic stability.

An interesting and important analysis was carried out by the authors of [28]. The authors attempted to reconcile the conflicting results obtained under different experimental conditions using NMR and radioactive tracer diffusion methods. It was concluded that the reason for most of the variation in the results of NMR experiments is the natural aggregation of erythrocytes in roulo (the coin column of erythrocytes). Part of the differences in the obtained data was due to the use of inappropriate mathematical approximations of the equations for two-site exchange, as well as differences in the time and conditions of the storage of blood samples. When the factors distorting the experimental data were corrected, it turned out that the results of measuring the diffusion of water molecules in erythrocytes using NMR methods and the diffusion of a radioactive tracer coincided in a rather narrow range of values. It turned out that the average “lifetime” of water molecules inside fresh normal human erythrocytes at room temperature (20–25 °C) was in the range from 9.8 to 14.0 ms. The range of the permeability of the erythrocyte membrane for water molecules was from 3.3 × 10^−5^ m/s to 4.7 × 10^−5^ m/s.

There have been attempts to use the method of PFG NMR for the study of various pathologies [29,30,31]. In [29], an analysis of the time dependence of the diffusion coefficient of water molecules in erythrocytes in PFG NMR experiments was carried out. It was demonstrated that the diffusion coefficient *D_p_* in the long-term mode of diffusion is highly dependent on the extracellular volume fraction. This explains the significant decrease in the *D_p_* value at the early stage of cerebral ischemia, where as soon as a few minutes after the ischemic stroke, the extracellular volume in the affected area of the brain decreases significantly. The addition of pCMBS led to a decrease in membrane permeability of 50% due to the blocking of water transport through protein channels, while the lipid pathways remained unchanged.

Another relevant work is related to the study of the effect of cholesterol on the permeability of erythrocyte membranes for water molecules [30]. Here, interest was due to the possibility of using the method of PFG NMR for the clinical diagnosis of liver diseases, hypo- or hyperc-holesterolemia, which affects the molar ratio of cholesterol to phospholipid (C/P) in the erythrocyte membrane. The authors concluded that, in human erythrocytes, the average intracellular “lifetime” of water molecules, which characterizes the rate of water transport under the conditions of equilibrium exchange, is a nonmonotonic function of the molar ratio of cholesterol to membrane phospholipids. A decrease in the C/P ratio in the membrane from an average value of 0.92 for normal cells (control) to a value of 0.46 had little effect on the average intracellular “lifetime” of water molecules and the permeability of the erythrocyte membrane. However, an increase in the content of cholesterol in the membrane had a noticeable effect on these parameters. At the ratio C/P = 1.5, the rate of the diffusion of water molecules decreased by 3.5 times. Moreover, a further increase in the cholesterol content to the ratio C/P = 1.9 led to an increase in the diffusion rate to the normal (control) value, which was characterized by a “lifetime” of 8–9 ms.

In [31], the PFG NMR technique was applied in combination with other methods to study the permeability of aquaporin-1 (AQP1)-deficient erythrocyte membranes for water molecules. Blood samples were obtained from people with a rare Coltonnull phenotype. These erythrocytes, completely devoid of AQP1 but devoid of other defects, are the membranes required for the direct measurement of parameters not mediated by AQP1 function. It was shown that AQP1 (>85%) makes a greater contribution to the permeability of the erythrocyte membrane for water molecules. The authors suggested that there are pathways in erythrocyte membranes other than AQP1 and diffusion through the lipid bilayer. After analyzing the dependence of *P* permeability in erythrocytes with complete AQP1 deficiency, they observed two diffusion pathways for water molecules: inhibited and non-inhibited by PCMBS.

**Figure 5:**
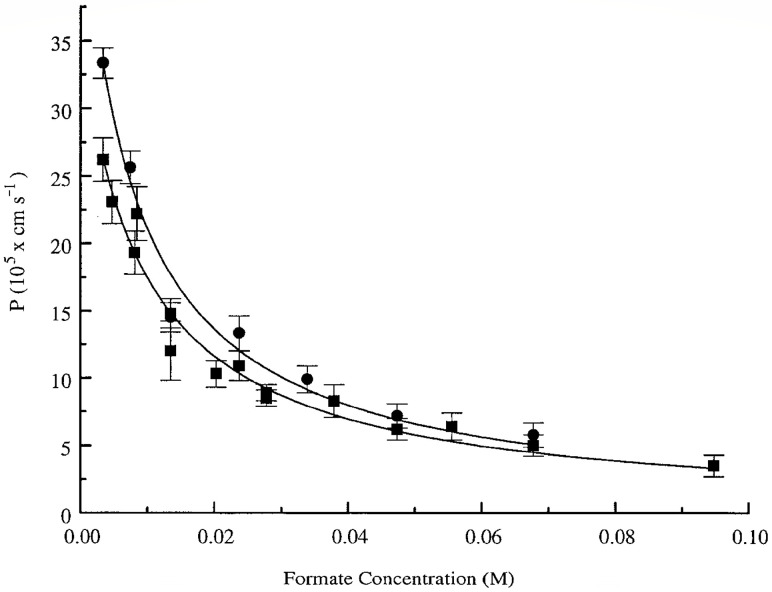
Concentration dependence of the formate membrane permeability coefficient in a suspension of RBCs. Squares denote *P*_formate_ obtained from the ^13^C-formate “manganese doping” method, and circles denote results obtained from PFGSE NMR experiments. The error bars in the main graph denote ±1 SD [32].

The authors of [32] evaluated the permeability of the erythrocyte membrane for formate using PFG NMR. Interest in this substance was due to several reasons: (1) formate is found in erythrocytes; (2) its signal in the ^1^H spectrum is represented by a singlet located separately from the proton signals of the system. As a result, in a suspension of erythrocytes in a 70 mM solution of sodium formate, from the data of the PFG NMR experiment, the permeability of the erythrocyte membrane *P* = 6.8 × 10^−7^ m/s was obtained. An analysis of the concentration dependence of the permeability of the erythrocyte membrane for formate molecules (Figure 5) showed a decrease in the *p* value from ≈3.6 × 10^−6^ m/s to ≈3.8 × 10^−7^ m/s with an increase in the concentration of the formate solution from 5 to 95 mM. Thus, it was shown that the exchange of formate under the conditions of chemical and thermodynamic equilibrium proceeds rapidly, but the concentration dependence of its rate can be studied by using the PFG NMR method.

Our team studied the permeability *P* of the erythrocyte membrane for water molecules and various C_60_ fullerene derivatives using the ^1^H PFG NMR method [33,34]. The latter turned out to be possible for compounds whose signals in the ^1^H NMR spectra do not overlap with the signals of the erythrocyte membrane components.

By investigating the permeability of the erythrocyte membrane for water molecules, the diffusion decays of the protons of water molecules were obtained at various temperatures (from 5 to 35 °C) in a suspension of erythrocytes [33]. For each temperature experiment, diffusion decays were obtained for different diffusion times td. Thus, a significant array of experimental data was obtained, the analysis of which was carried out in accordance with the theory described in Section 2. Below, some illustrations of the results obtained are given.

**Figure 6:**
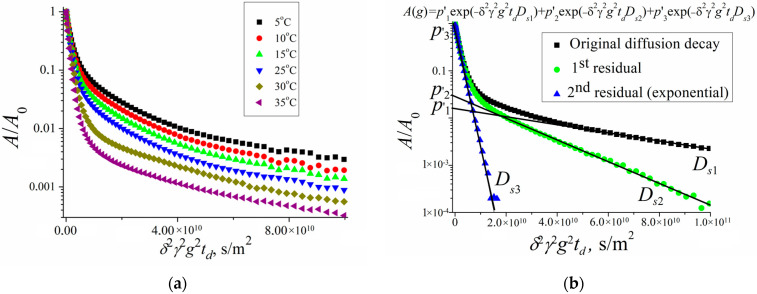
(**a**) Diffusion decays of protons of water molecules in a suspension of erythrocytes at different temperatures (temperature values are given in the inset, *t_d_* = 20 ms). (**b**) The procedure of decomposition of the original diffusion decay on three exponential components according to Equation (2) is shown: *D_s_*_3_ = 0.60·10^−9^ m^2^/s, *p*_3_′ = 0.947; *D_s_*_2_ = 0.76·10^−10^ m^2^/s, *p*_2_′ = 0.045; m^2^/s; *D_s_*_1_ = 1.37·10^−11^ m^2^/s, *p*_1_′ = 0.008 (1—original diffusion decay, 2—1st residual, 3—2nd residual). Reprinted with permission from [33].

Figure 6 shows the diffusion decays of the protons of water molecules in a suspension of erythrocytes at different temperatures and an example of diffusion decay decomposition in accordance with Equation (2). Figure 6a shows diffusion decays at diffusion time *t_d_* = 20 ms. With an increase in temperature, a change in the slope of the “tail” in diffusion decays is observed, which, as is shown below, indicates a change in the permeability of the erythrocyte membrane with a change in temperature. Figure 6b clearly shows the principle of the decomposition of the diffusion decay into components characterized by partial self-diffusion coefficients (SDC) *D_si_* with the corresponding populations *p_i_*. The decomposition was carried out by the sequential subtraction of the “tail” of diffusion decay. The presented decomposition into three components made it possible to distinguish three types of water in the erythrocyte suspension: intracellular (*D_s_*_1_, *p*_1_), intercellular (*D_s_*_2_, *p*_2_), and bulk (*D_s_*_3_, *p*_3_).

The paper [33] presents a detailed analysis of the obtained experimental data. It should be noted that the populations *p*_1_ and *p*_2_ and the SDC *D_s_*_1_ and *D_s_*_2_ depend on the diffusion time *t_d_*. Information about the permeability of the erythrocyte membrane for water molecules and cell size is obtained from data on the self-diffusion of intracellular water, the behavior of which is characterized by *D_s_*_1_ and *p*_1_.

The obtained experimental dependence of the SDC *D_s_*_1_ of intracellular water molecules on the diffusion time *t_d_* turned out to be flatter than $t_{d}^{-1}$. This is explained by the existence of membrane permeability. Therefore, the application of Equation (3) to calculate the cell size *a* turned out to be possible only after applying the scaling approach in accordance with Equation (4). As a result, the dependence $D_{s}^{eff}\left(t_{d}\right)$ was obtained, which does not depend on temperature. This led to the conclusion that the size of the diffusion restrictions of water molecules *a* (the size of an erythrocyte cell) does not change with temperature. The diffusion limiting size *a* was 2.1 μm.

The value of the permeability *P* of the erythrocyte membrane, calculated in accordance with Equation (5), changes from 0.3·10^−5^ to 0.5·10^−5^ m/s with an increase in temperature from 5 to 35 °C. The measured values of the permeability of the cell walls of erythrocytes are lower than the values obtained by the authors of [28]. This difference can be explained by the fact that, in [28], spin-echo decay was observed when spin-echo signals decreased within one order of magnitude *A*. This fact did not allow for a correct measurement of the self-diffusion coefficient *D_s_*_1_, which characterizes the intracellular water component. In the proposed work, the diffusion decays of the water molecules were analyzed within two and three orders of magnitude, which make it possible to estimate *D_s_*_1_ more accurately.

Due to the permeability of erythrocyte membranes, there is an exchange between bulk, intercellular, and intracellular water. To calculate the residence time of a water molecule *τ* inside the cell, we used the two-component model of molecular exchange described in Section 2. We analyzed the dependence of the population of intracellular water *p*_1_ on the diffusion time *t_d_* after subtracting the spin–lattice relaxation component. The value of the lifetime *τ* of the molecule inside the cell was 20 ± 2 ms at 30 °C. The obtained value of *τ* is close to the results obtained in [23]. However, it turned out to be higher than that in [28], which can be explained by a different sample temperature being used in the experiment (20–25 °C).

**Figure 7:**
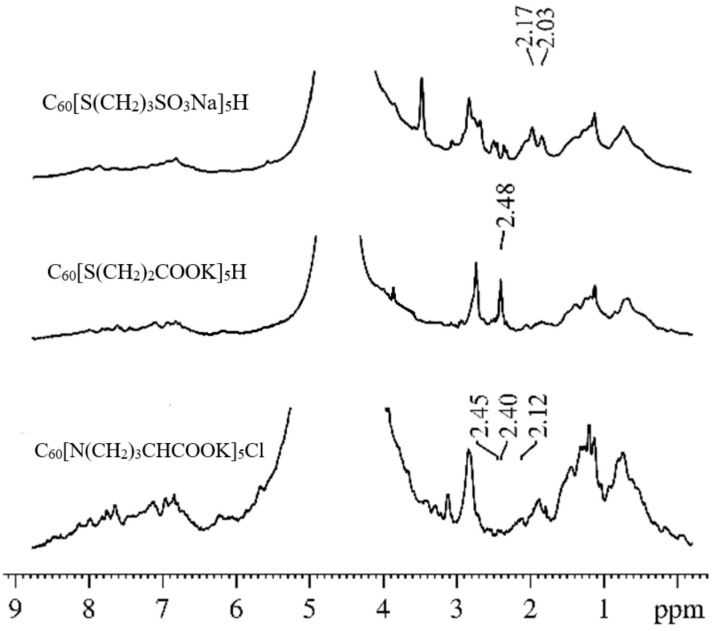
The ^1^H spectra of the RBC suspensions with added C_60_ fullerene derivatives. Reprinted with permission from [32].

In [35], we presented the results of studies of the mobility of C_60_ fullerene derivatives (C_60_[S(CH_2_)_3_SO_3_Na]_5_H, C_60_[S(CH_2_)_2_COOK]_5_H, and C_60_[N(CH_2_)_3_CHCOOK]_5_Cl) molecules in an erythrocyte suspension. Using the analysis of the ^1^H spectra at different amplitudes of the magnetic field gradient, we showed that the signals from the protons of the studied compounds can be unambiguously identified in the spectra of suspensions. These signals do not overlap with the proton signals of the membrane components. Examples of the ^1^H spectra of the systems under study (erythrocyte suspensions with the addition of compounds of C_60_ fullerene derivatives) are shown in Figure 7. The chemical shifts of ^1^H compounds change slightly in erythrocyte suspensions compared to solutions due to the interaction of the C_60_ fullerene derivative with erythrocytes.

The isolated ^1^H signals of the molecules of the studied compounds in the spectra made it possible to correctly register the diffusion decays of the molecules of C_60_ fullerene derivatives in a suspension of erythrocytes. From an analysis of the experimental data in accordance with Equation (2), the values of the self-diffusion coefficients of the molecules of C_60_ fullerene derivatives in a suspension of erythrocytes were obtained. It was concluded that, in the suspension of erythrocytes, some molecules of C_60_ fullerene derivatives are bound to the cell membrane, and others are in the aqueous phase in the form of isolated and associated molecules. An analysis of the dependence of the population of slowly moving molecules of fullerene derivatives *p*_1_ on the diffusion time *t_d_* made it possible to estimate the lifetime *τ* of the molecules of C_60_ fullerene derivatives in the erythrocyte membrane from Equation (6). The lifetimes *τ* for C_60_ fullerene derivatives are given in Table 1.

Another direction in the method of PFG NMR is diffusion–diffraction NMR, which can be used to characterize the shape and size of erythrocytes [36,37,38,39,40]. In its simplest form, the PGSE experiment consists of an ordinary spin-echo pulse sequence in which two magnetic field gradient pulses of magnitude *g* and duration *δ* are applied on either side of the 180º refocusing pulse separated by time ∆ [37]. In the limit *δ* → 0 while *g* → ∞, known as the short gradient pulse approximation, the wave vector *q*, defined as *q* ≡ *γgδ*/2π, where *γ* is the magnetogyric ratio, is finite, and the echo attenuation is given by

(7) $$E\left(q,∆\right)=\iint \rho \left(r_{0}\right)P\left(r_{0}|r,∆\right)\times exp\left[i2\pi q\cdot \left(r-r_{0}\right)\right]drdr.$$

In Equation (7), *P*(*r*_0_|*r*,∆) is the propagator that determines the probability of a molecule, initially at position *r*_0_, being found at position *r* after time ∆, where ρ(*r*_0_) is the initial density of the molecules. For free isotropic diffusion, the propagator is Gaussian, and the integration in Equation (7) yields the following expression relating the echo intensity to the diffusion coefficient *D*:

(8) $$E=\frac{A_{g}}{A_{0}}=\exp(-\left(2\pi g{)}^{2}∆D\right),$$

where *A*_0_ is the amplitude of the signal when *g* = 0, and *A_g_* is the amplitude when *g* ≠ 0.

**Figure 8:**
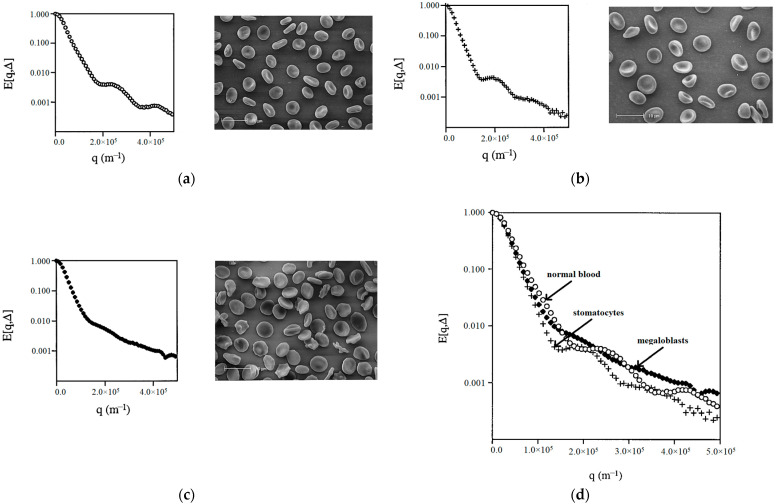
Diffusion–diffraction of water in suspensions of normal and abnormal erythrocytes with corresponding electron micrographs: (**a**) normal blood; (**b**) stomatocytes; and (**c**) megaloblasts. For comparative purposes, the three q-space plots were superimposed in (**d**) [36].

The diffusion-coherence phenomena described above can be visualized as a series of maxima and minima when the relative signal intensities from the PGSE NMR spectra are plotted as a function of q in a *q*-space plot. *q*-Space can be thought of as the reciprocal space, in the Fourier transform sense, of spin displacement in the characteristic time of the experiment. Diffusion interference is manifested as a shoulder in the initial part of the attenuation curve and diffusion–diffraction as the subsequent series of maxima and minima (see Figure 8).

The authors of the work [36] applied the NMR diffusion–diffraction technique to characterize the shape of erythrocytes in suspensions containing erythrocytes with different shapes/forms, prepared by varying the conditions of the suspension medium, such as osmolality, and changing the metabolism to affect the concentration of adenosine triphosphate. As an example of the practical application of this approach, abnormal erythrocytes were also examined in patients with hereditary stomatocytosis and megaloblastic anemia (Figure 8).

The experimental results for erythrocytes with different shapes/forms showed that each erythrocyte shape can be assigned a “characteristic” q-space plot/profile. It was found that the uniformity of the shape and/or size of cells is an important factor influencing the intensity of the diffusion–diffraction peaks. The diffusion–diffraction pattern obtained for stomatocytes (Figure 8a) was similar to that obtained for normal cells (Figure 8b): two well-defined diffraction peaks were observed. However, a more detailed examination showed that the positions of the two diffraction minima for stomatocytes are shifted towards much smaller values (Figure 8d). At the same time, megaloblasts did not give a clear diffusion–diffraction pattern; only one poorly expressed diffraction peak was observed (Figure 8c). The absence of a clear diffusion–diffraction profile of megaloblasts on the q-space plot indicated heterogeneity in either the shape or size of the cells. This sensitivity of the method is important for its further application.

Further development of this direction is presented in [37,38]. The q-space plots obtained from PFG NMR diffusion experiments in an erythrocyte suspension and in similar computer-simulated cell suspensions demonstrate complex biophysical profiles of transport processes and microstructures characterizing cell data [37]. The set of coarse and fine features that make up each individual profile can potentially give a lot of detail describing the system. These include membrane permeability coefficients; hematocrit; diffusion coefficients; cell packing; cell sizes; and cellular geometry. Further, additional information was obtained on the measurement and analysis of diffusion-coherence phenomena on q-space plots from erythrocyte suspensions [38]: (1) q-positions of diffraction peaks depend on the observation time, especially in the mode of unlimited diffusion; (2) the measured or apparent RMS displacement has a non-linear dependence on the membrane permeability.

The use of NMR diffusion–diffraction in suspensions of diamagnetic erythrocytes made it possible to obtain and analyze q-space plots for water molecules [39]. Several important conclusions were made: (1) the shape of the q-space plots depends on the direction along which diffusion is measured, which suggests cell alignment in the magnetic field of the NMR spectrometer; (2) diffusion anisotropy changes in a predictable way due to the transfer of hemoglobin to a paramagnetic form; (3) the shapes of the q-space graphs change in a predictable way due to the suppression of water transfer; and (4) cell diameter and intercellular distance can be measured by the positions of interference minima and maxima on q-space graphs.

In [40], to study the effect of erythrocyte cell geometry on biochemical and energy processes in cells, erythrocytes were incorporated into elastic gelatin hydrogels. This created conditions for the controlled elongation of their normal discocytic shape in all orientations. When stretching or compressing the gels containing erythrocytes, the changes in cell morphology were studied using ^1^H-PFG NMR spectroscopy. Measurements of the apparent diffusion coefficient of water in three orthogonal directions revealed a tunable anisotropy in the environment of the hydrogel samples.

## 4. Research on Other Biological Systems

The application of the PFG NMR method is not limited to the study of erythrocytes [41,42]. In this section, we present works devoted to the study of other cellular systems.

In the previous section, the results of studies of the permeability of erythrocyte membranes for various substances are presented. However, the difficulty in working with such systems is the multiple signals of the membrane components in ^1^H NMR spectra. While the signal from water molecules can be unambiguously identified and isolated, for other molecules, the ^1^H signals often overlap with the signals from the protein or lipid part of the erythrocyte membrane. An attempt can be made to solve this problem by using the ghosts of erythrocytes. The ghosts of erythrocytes are membrane shells of erythrocytes, obtained as a result of the release of hemoglobin from cells in a hypotonic saline solution.

The authors of [41] used erythrocyte ghosts as a model for studying the effects of compartmentation on the MR signal from biological tissues. The application of this model made it possible to control the biophysical parameters that affect the multicomponent signals from the cellular system. The effect of cell density on the measurement of the diffusion of water molecules in the system was studied. It turned out that an increase in cell density from 17 to 67% does not significantly influence the residence time *τ* of a water molecule inside the cell. At the same time, different values of the “lifetime” *τ* of intracellular water were obtained using two different models used for processing experimental data: a biexponential model and a two-compartment model that accounted for exchange between compartments. In the first case, the value of *τ* was in the range from 20.5 ± 0.2 to 23.7 ± 0.2 ms in the specified range of cell concentrations; in the second case, it was from 10.0 ± 0.7 to 16.1 ms. The bi-exponential fitting of the data turned out to be under-parameterized, since the diffusion coefficients and the relative fractions of the fast and slow components depended on the experimental data acquisition parameters, in particular, on the diffusion time. This method of analysis is not suitable for erythrocyte ghosts due to compartmental water exchange, but it may be adequate for tissues in which water exchange between compartments is slow, i.e., tissues with large cells, a low membrane permeability to water, and a lower speed. Both methods of analysis proved to be effective in tracking the changes in the ghost model when it was perturbed. This has been demonstrated in experiments with cell density variation, cell swelling and shrinkage, and a reduction in membrane water permeability using a water channel blocker (pCMBS).

We also used the ghosts of erythrocytes to assess the permeability of the membrane for the fullerene derivative C_60_ C_60_[S(CH_2_)_3_SO_3_Na]_5_H [42]. In this work, we compared the results of experiments performed in a suspension of erythrocytes, ghosts of erythrocytes, and phosphatidylcholine liposomes. In the ^1^H NMR spectrum of an aqueous suspension of erythrocyte ghosts with an added C_60_ fullerene derivative, there were no signals belonging to the protein component of the erythrocyte. Fullerene derivative molecules were absorbed by erythrocyte ghosts and phosphatidylcholine liposomes as manifested in the self-diffusion coefficients of (7.9 ± 1.2)·10^−12^ m^2^/s and (7.7 ± 1.2)·10^−12^ m^2^/s, which are also close to the lateral diffusion coefficients of (6.5 ± 1)·10^−12^ m^2^/s and (8.5 ± 1.3)·10^−12^ m^2^/s, respectively.

**Figure 9:**
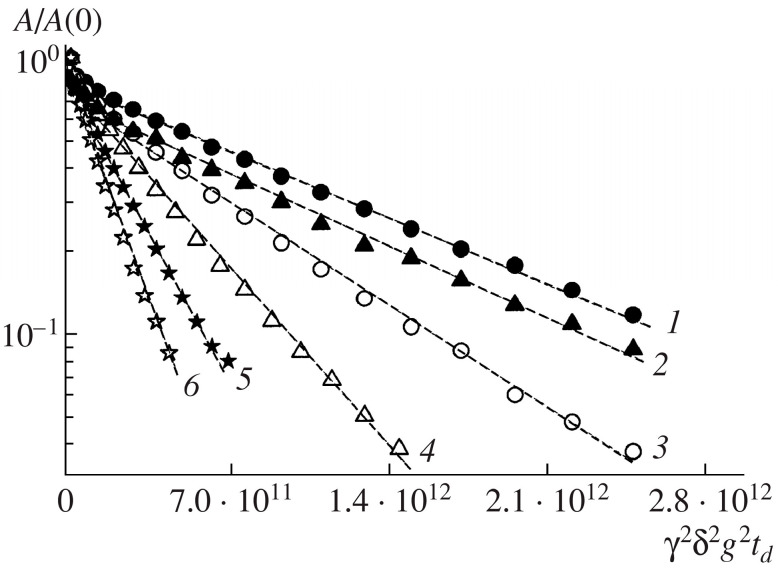
Water diffusion decay across bilayers at (*1*) 20, (*2*) 25, (*3*) 30, (*4*) 40, (*5*) 50, and (*6*) 60 °C. [45].

In addition to the ghosts of erythrocytes, lipid bilayers can be used as model systems for studying membrane permeability via PFG NMR [43,44,45]. For example, ref. [43] used stacks of macroscopically oriented fully hydrated bilayers of dimyristoylphosphatidylcholine, and in [44], dimyristoylphosphatidylcholine (DMPC) and dioleoylphosphatidylcholine (DOPC) were used to study the diffusion of water molecules. In such systems, the diffusion of water molecules is anisotropic and depends on the interaction of water molecules with hydrophilic heads of lipids and diffusion through lipid bilayers (transbilayer diffusion) and through lipid “cracks”. “Cracks” are located throughout the depth of the two-layer system, and the water entering them is characterized by relatively free self-diffusion. Careful hydration of the sample at the preparation stage gives the possibility to make the bilayers more extended with a minimum proportion of “cracks”. The transbilayer diffusion of water is the most interesting phenomenon, since it is related to the permeability of the bilayers. It can be obtained from diffusion decays obtained for bilayers oriented perpendicular to the magnetic field. In the paper [45], the temperature dependence of the self-diffusion coefficient of water through plane-parallel multibilayers of the phospholipid dioleoylphosphatidylcholine oriented on a glass substrate in the temperature range of 20–60 °C was studied. The diffusion decays of the protons of water molecules in the direction along the normal to the bilayer, obtained for the systems under study, are shown in Figure 9. The dotted line shows the regions corresponding to transbilayer diffusion. These regions were well described by Equation (1) and were characterized by one diffusion coefficient at each temperature.

The values of the coefficients of the transbilayer diffusion of water are 4 decimal orders less than those of bulk water, and they are 10 times less than the values of the coefficients of lateral diffusion of lipid under the same conditions. The temperature dependence of the diffusion coefficient is described by the Arrhenius law with an apparent activation energy of about 41 kJ/mol, which is much higher than the activation energy for bulk water diffusion (18 kJ/mol).

The PFG NMR technique has been successfully applied to the study of other cellular systems [17,18,46,47,48,49,50]. In [46], unique data on the diffusion of phosphorus-containing metabolites on ^31^P nuclei in the skeletal muscle of a goldfish (Curussius uurutus) were obtained. The authors measured the self-diffusion coefficients of ATP and creatine phosphate, the values of which were (2.13 ± 0.16)·10^−11^ m^2^/s and (3.28 ± 0.18)·10^−11^ m^2^/s, respectively.

In [47], the water permeability of the Gram-positive bacteria Corynebacterium glutamicum was assessed. From the data on the intracellular diffusion of water molecules, the value *p* = (4.8 ± 0.4)·10^−5^ m/s was obtained. According to the authors, this value is about 100 times higher than the ethanol permeability of Zymomonas mobilis.

In [17], the permeability of yeast cell membranes for water molecules at different times of cell growth was measured. The authors identified three types of water in the system: bulk, extracellular, and intracellular. It was shown that cell size *a* and cell membrane permeability *P* change depending on the growth phase of the yeast cell: the cell size increased and the permeability decreased with increasing growth time. The cell membrane permeability *p* values were 6.3·10^−6^, 8.4·10^−7^, and 1.5·10^−6^ m/s for cells incubated for 9 h (exponential growth phase), 24 h (end of exponential growth phase), and 48 h (stationary growth phase), respectively.

In [18], the permeability of the chlorella membrane for water molecules was estimated. From the data obtained during the PFG NMR experiment, in accordance with the theory described in Section 2, the cell size of chlorella *a* = 3.4 μm and the membrane permeability *P* = 10^−6^ m/s were estimated.

The authors of [48] measured the coefficients of the self-diffusion of water molecules in baker’s yeast by using PFG NMR with a variable pulse length gradient. The authors proposed a new protocol for the PFG NMR experiment for the coefficient of the local self-diffusion of water contained in living cells.

One of the latest reviews [49] details the results of PFG NMR experiments in the study of the diffusion of water molecules in plant roots. This review presents the results of studies of water transfer in roots along the symplastic system, from cell to cell through intercellular contacts with plasmodesmata, through aquaporins, and under the influence of changes in external pressure and of the composition of the gaseous atmosphere.

Our team studied the processes of water exchange in the cells of the yeast *Saccharomyces cerevisiae* races Y-3137 and Y-3327 at different growing times [50]. The diffusion decays of the protons of water molecules were recorded and analyzed in accordance with the theory described in Section 2. Table 2 presents the obtained results.

The higher permeability of the cell membrane of the yeast *Saccharomyces cerevisiae* Y-3327 explains its ability to withstand increased osmotic pressure and effectively ferment grain wort with a higher concentration of dissolved solids.

## 5. Lateral Diffusion Studies

In addition to studying the processes of molecular metabolism in cells, the PFG NMR method can be used to study the lateral diffusion of lipid molecules [41,48,49,50,51,52]. The relevance of such work is due to the fact that lipids form the basis of the biological membrane and are directly responsible for the integrity of the cell and its communication with the external environment. A special place is occupied by the study of lipid rafts–domains in the lipid bilayer with an increased concentration of cholesterol, sphingolipids, and some types of proteins [51]. These sites coordinate cellular processes, affect the fluidity of the biomembrane, serve as organizing centers for the assembly of signaling molecules, regulate the movement of membrane proteins, etc. Thus, the assessment of the effect of lipid composition (cholesterol, in particular) on the heterogeneity of the structure and dynamics of molecules in lipid bilayers is relevant for understanding the functioning of biomembranes. As practice has shown, it is better to use oriented bilayers to perform NMR experiments to study the lateral diffusion of lipids.

An evaluation of the effect of cholesterol on the lateral diffusion of phospholipids in the oriented bilayers of dimyristoyl phosphatidylcholine (DMPC), sphingomyelin (SM), palmitoyl oleoylphosphatidylcholine (POPC), and dioleoyl phosphatidylcholine (DOPC) with the addition of cholesterol (CHOL) up to 40 mol·% was carried out in [52]. The obtained coefficients of the lateral diffusion of the lipids in these systems made it possible to distinguish liquid-ordered (l_o_) and liquid-disordered (l_d_) phases in lipid–cholesterol systems. The authors showed that liquid domains with a high molecular order are formed within a single lipid bilayer. Further, ref. [53] showed that saturated lipids, such as l,2-dipalmitoyl-sn-glycero3-phosphocholine (DPPC) and chicken egg yolk sphingomyelin (eSM), form more ordered phases than unsaturated lipids, and the addition of CHOL significantly enhances the ordering, especially for lipids with saturated chains. Experimental data have demonstrated that DPPC and eSM prefer to be in an ordered phase, while DOPC prefers an unordered phase. Another significant result was that CHOL is divided into both phases (ld and lo) to approximately the same extent. This indicates that CHOL does not give special preference to any of these phases, and there are no specific interactions between CHOL and saturated lipids. Further work in this field [54] showed that lateral diffusion is the same for all components, regardless of molecular structure (including CHOL), if they are in the same domain or phase in the membrane. Research [55], performed in bilayers prepared from a mixture of the lipids DOPC, CM, and CHOL, showed that the system forms a lamellar liquid-crystal phase, and in a certain temperature range and cholesterol concentrations, the system is divided into two subphases, namely, disordered l_d_, enriched with DOPC, and ordered l_o_, enriched with CM, each of which is characterized by its own value of the lateral diffusion coefficient, which differs by 1.5–5 times. An analysis of the dependence of the lateral diffusion coefficient in phases on the concentration of cholesterol showed that, in the LC-ordered phase, the lateral diffusion coefficient increases in the concentration range of 15–35 mol·% CHOL. The authors explain this behavior by the redistribution of lipid components with an increase in the concentration of CHOL in the system, which eventually leads to an increase in the concentration of DOPC in the l0 phase.

In addition to the studies of cholesterol as a compound affecting the properties of lipid bilayers, other molecules that occur in biological systems and that can influence biomembranes are also considered [44,56,57,58].

In [56], the influence of a number of amphiphilic esters of 1,1-dimethyl-3-oxobutylphosphonic acid (diethyl, dipropyl, and dibutyl) with a regular change in the number of CH_2_- groups in the hydrocarbon (hydrophobic) part at ester concentrations up to 30 mol·% on the lateral diffusion of molecules of the lipid dioleoylphosphatidylcholine was examined. It was concluded that the presence of esters does not affect the phase state of the system: the lamellar liquid-crystalline phase is retained; the ester molecules are incorporated into the bilayer and have the same orientation as the phospholipid molecules. The authors found that the lateral diffusion coefficients of the lipid in the presence of esters are increased compared to the lipid bilayer in the absence of phosphonic acid esters and have a monotonic dependence on the number of CH_2_- groups in the ester molecule. The most probable site for the incorporation of amphiphilic ester molecules is the interphase region located between the hydrophilic and hydrophobic parts of the bilayer; the incorporation of molecules into the interphase leads to the disordering of the bilayer and an increase in the lateral diffusion of lipids and the permeability of the bilayer compared to the bilayer without esters.

In [44], the effect of polyacrylic acid (PAA) on the lateral diffusion of lipids in DOPC-PAA and DOPC-PAA bilayers was evaluated. It was found that the effect of polyacrylic acid depends on the pH of the medium. In an alkaline medium, no dependence of the lateral diffusion coefficient on the PAA concentration was observed. In an aqueous medium with pH = 5.9 ÷ 4.6, a decrease in the lateral diffusion coefficient of lipids was observed with an increase in PAA concentration.

**Figure 10:**
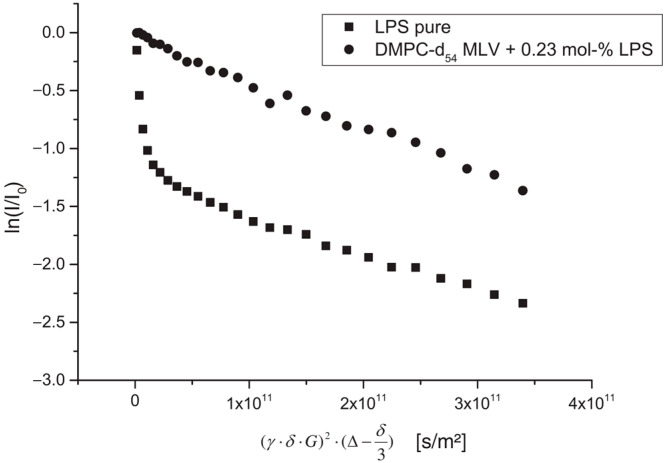
Stejskal–Tanner plots obtained from ^1^H PFG-NMR measurements on sonicated solutions of pure LPS (squares) and a DMPC-LPS mixture (circles) [57].

The authors of [57] studied the effect of lipopolysaccharide (LPS) from Escherichia coli on the structure, dynamics, and mechanical strength of phospholipid membranes formed from 1,2-dimyristoyl-sn-glycero-3-phosphocholine (DMPC). A comparison of the results of the PFG NMR experiments for aqueous solutions of LPS and the DMPC-LPS system (an example of diffusion decay for each system is shown in Figure 10) made it possible to establish that, in aqueous solutions, LPS molecules are in the form of partially associated molecules with *r* = 5.8 nm and large vesicles with *r* = 80 nm. When LPS is added to DMPC, all LPS mobile states disappear, which is explained by the integration of LPS molecules into the bilayer.

In [58], the effect of curcumin on the DMPC/cholesterol bilayer was studied. It has been shown that curcumin at concentrations up to 10 mol·% somewhat reduces the lateral diffusion of phospholipids in bilayers without cholesterol, but in the presence of 5–33 mol·% of cholesterol in dimyristoylphosphatidylcholine bilayers, the effect of curcumin is very small, since, in direct competition for the insertion of a lipid bilayer into DMPC during the formation of a bilayer from an initially homogeneous mixture, cholesterol overwhelmingly wins and, therefore, inhibits the incorporation of curcumin.

## 6. Conclusions

This paper presents the main results of the application of the pulsed field gradient NMR (PFG NMR) method in the study of various biological systems. This paper discusses the basic ideas surrounding the theory of the PFG NMR experiment and the features of processing experimental data obtained for permeable systems (biological cells and lipid bilayers). An analysis of the literature data showed that the PFG NMR method can be used to assess the permeability of biological membranes for water molecules and other substances. From the PFG NMR experiment, such parameters as the coefficients of the self-diffusion of diffusant molecules and the time of their residence inside the cells can be determined. A significant number of works are devoted to the study of the permeability of the membrane of erythrocytes, which are the main component of blood and perform a number of important functions, for water molecules. The signals of the membrane components in the NMR spectra complicate the analysis of diffusion data for molecules of biologically active substances. This problem can be solved by using erythrocyte ghosts or model lipid bilayers. The use of the latter systems makes it possible to study the lateral diffusion of lipid molecules in bilayers. The obtained results make a significant contribution to understanding the functioning of biological cells.

## Figures and Tables

**Table 1:** The self-diffusion coefficients *D_s_*_1_; population *p*_1_ at *t_d_*→0, *p*_1_(0); and lifetimes *τ* of fullerene derivatives molecules in erythrocytes [35].

Compound	*D_s_*_1_^s^·10^12^, m^2^/s	*p*_1_(0)	*τ*, ms
C_60_[S(CH_2_)_3_SO_3_Na]_5_H	5.5 ± 0.8	0.33	440 ± 70
C_60_[S(CH_2_)_2_COOK]_5_H	5.0 ± 1.0	0.13	470 ± 70
C60[N(CH2)3CHCOOK]5Cl	6.0 ± 1.0	0.06	1200 ± 300

**Table 2:** Yeast cell size *d* (diameter) and permeability *P* at different times of cell cultivation.

Race of Yeast	Cultivation Time, h	Cell Diameter *d* Calculated from NMR Data, µm	Permeability *P* × 10^−6^, m/s
Y-3137	24	7.9	0.29
	48	7.9	0.61
Y-3327	24	5.1	0.19
	48	3.8	0.53
